# Estimated prevalence rates and risk factors for common mental health problems among Syrian and Afghan refugees in Türkiye

**DOI:** 10.1192/bjo.2022.573

**Published:** 2022-09-15

**Authors:** Gulsah Kurt, Peter Ventevogel, Maryam Ekhtiari, Zeynep Ilkkursun, Merve Erşahin, Nuriye Akbiyik, Ceren Acarturk

**Affiliations:** Department of Psychology, Koc University, Istanbul, Türkiye; Public Health Section, United Nations High Commissioner for Refugees, Geneva, Switzerland; Department of Sociology and International Relations, Koc University, Istanbul, Türkiye; Department of Clinical Psychology, Erasmus University Rotterdam, The Netherlands; Faculty of Humanities and Social Sciences, University of Bergamo, Italy

**Keywords:** Syrians, Afghans, refugees, mental health, Türkiye

## Abstract

**Background:**

Türkiye hosts 4 million refugees and asylum seekers, with Syrians and Afghans being among the largest refugee groups in country. There are limited comparative data on the conflict- and displacement-related experiences of these groups and the relation with mental health status.

**Aims:**

To assess the mental health status of Syrians and Afghans in Türkiye, identify risk factors and explore to what extent differences in mental health conditions are related to potentially traumatic events and post-displacement stressors.

**Method:**

Two parallel online survey studies were conducted between April and June 2021 among 798 Syrians and 785 Afghans in Türkiye. Data were collected on sociodemographic characteristics, traumatic events (Harvard Trauma Questionnaire), post-displacement stressors (Post-Migration Living Difficulties Checklist), symptoms of depression and anxiety (Hopkins Symptoms Checklist-25) and post-traumatic stress disorder (PTSD) (Post-Traumatic Stress Disorder Checklist for DSM-5, short form).

**Results:**

For Syrian and Afghan participants respectively, estimated prevalence rates were: 41.1% and 50.3% for depression; 39.6% and 41% for anxiety; and 41.6% and 46.5% for PTSD. In both groups, significant predictors were female gender, exposure to potentially traumatic events, and structural and socioeconomic post-displacement stressors. Additional risk factors were older age for Afghans and higher education for Syrians.

**Conclusions:**

Self-reported symptoms of common mental health problems are highly prevalent among Syrian and Afghan refugees and associated with a wide range of risk factors. After controlling for conflict- and displacement-related experiences, Afghans reported higher anxiety symptoms than Syrians, which is likely related to their legal status in Türkiye.

Violence, persecution, economic and political upheavals have led to an unprecedented increase in forced displacement. By mid-2021, the world had 26 million refugees, with Syrians and Afghans among the largest groups.^1^ In several decades, Türkiye has become an important transit and destination country, currently hosting 4 million refugees and asylum seekers.^2^ The largest group in Türkiye are Syrians (3.7 million), who have been seeking refuge from civil unrest since 2011, but the country also hosts over 125 000 Afghans, who have been fleeing their war-torn country for over 40 years.^3^ Most Afghans flee to neighbouring countries such as Iran and Pakistan,^1^ but Türkiye plays a significant role in the onward movement of Afghan refugees towards the Global North.^2,4^ They are currently the second largest asylum-seeking group in Türkiye.^5^ The situation in Afghanistan deteriorated dramatically in 2021, resulting in the ousting of the government and a volatile sociopolitical situation causing increasing numbers of Afghan refugees to flee to neighbouring countries.^6^ The fragile situation will likely lead to new refugee movement of Afghans in the near future, with many having an increased risk of developing mental health problems.^7^ Compared with Syrians, Afghan refugees are relatively understudied. The massive influx of Syrian refugees to neighbouring countries has attracted significant scholarly attention to their mental health and their displacement conditions,^8,9^ but there has been less of such research on Afghan refugees in the Middle East and Türkiye.^10^

Many Syrians and Afghans have experienced severe potentially traumatic events such as witnessing atrocities, prolonged deprivation and the loss of loved ones either in their home country or en route to destination countries, all of which negatively affect their mental health and well-being and may undermine their adaptive abilities.^11,12^ The effects of accumulated adverse experiences are compounded by factors in the displacement settings, as ongoing and proximal stressors such as economic, social and legal difficulties exacerbate the risk of developing mental health problems.^13^ Both in neighbouring and Western countries, displaced Syrians and Afghans have reported a range of displacement-related difficulties, including lack of access to the labour market and basic services, linguistic and cultural differences, social isolation, discrimination, and long and complex asylum procedures.^10,12,14^ On top of these difficulties, the COVID-19 pandemic pushed them further to the edges.^15^ Mental health problems are two to three times higher in refugees and conflict-affected populations,^16^ yet knowledge on differences between refugee populations is limited.^17^ There are substantial differences in the legal status of Syrian and Afghan refugees in Türkiye. Syrians have been granted a ‘temporary protection status’ which provides them with certain rights that regular asylum seekers do not have, such as access to the labour market, healthcare services and education.^18^ Afghans have to follow the regular procedures to apply for asylum, as described in the 2013 Law on Foreigners and International Protection. However, because of the prolonged and uncertain decision-making process, many Afghans do not apply for asylum in Türkiye but live as irregular immigrants.^4^ These differences in residency status between Afghans and Syrians are likely to lead to more precarious living conditions among Afghans, which can take toll on their mental health. To our knowledge, no study has systematically investigated and compared the displacement-related experiences and mental health problems of Syrian and Afghan refugees in Türkiye.

The present study aims to address this gap and provide insight into mental health conditions among Syrians and Afghans in Türkiye. Given the protracted humanitarian crisis in Afghanistan and legal barriers for Afghans in Türkiye, we hypothesised that compared with Syrians, Afghans would report a higher level of potentially traumatic experiences and have more post-displacement stressors, and that this would be associated with higher self-reported common mental health problems.

## Method

### Study design and participants

We conducted two parallel online survey studies between April and June 2021, using convenience sampling to recruit participants via three local non-governmental organisations and a municipality working with Syrians and Afghans. These organisations advertised the study on their social network sites (Facebook, Telegram, WhatsApp, etc.) The inclusion criteria were (a) being 18 years or older, (b) migration to Türkiye due to the conflicts in Syria or Afghanistan and (c) being literate in the Arabic or Dari language. Participants filled out the survey on the Qualtrics online survey platform. Written informed consent was obtained from all participants at the beginning of the survey. Those who agreed to participate were invited to complete an online questionnaire in Dari or Arabic. Completion of the questionnaire took around half an hour. Participants were reimbursed with a grocery voucher for US$3.75 for their time. The authors assert that all procedures contributing to this work comply with the ethical standards of the relevant national and institutional committees on human experimentation and with the Helsinki Declaration of 1975, as revised in 2008. All procedures involving human participants/patients were approved by the ethics committee of Koc University, Istanbul, Türkiye (approval number of 2020.423.IRB3.161). We used the existing Arabic and Dari versions of all the scales except for the one measuring post-displacement stressors. Following the World Health Organization guidelines,^19^ the scale was translated and back-translated by different bilingual speakers. Any inconsistencies between the original and back-translated document were resolved by referring to the original version.

The present sample consisted of 798 Syrian (467 of whom (58.5%) were female) and 785 Afghan refugees (265 (33.8%) female) living in Türkiye with an average age of 33 years (s.d. = 10.6) and 30 years (s.d. = 9.5) respectively. Most Syrian participants were married (598, 74.9%), had at least one child (654, 82%) and were under temporary protection status (713, 89.3%). Of the Afghan participants, 49.9% (392) were married, 50.3% (395) had at least one child and 55.2% (433) were asylum seekers. The average length of stay was 69 months (s.d. = 26.35) for Syrians and 36.3 months (s.d. = 24.43) for Afghans. Table 1 summarises the sample characteristics.

**Table 1:** Sample characteristics (*n* = 1583)

	Syrian participants (*n* = 798)	Afghan participants (*n* = 785)
Gender, female: *n* (%)	467 (58.5%)	265 (33.8%)
Age, years: mean (s.d.)	33.22 (10.62)	29.60 (9.50)
Marital status, *n* (%)		
Married	598 (74.9%)	392 (49.9%)
Unmarried	116 (14.5%)	349 (44.5%)
Divorced	24 (3%)	27 (3.4%)
Widowed	60 (7.5%)	17 (2.2%)
Children (yes), *n* (%)	654 (82%)	395 (50.3%)
Income, *n* (%)		
<1000 Turkish liras	266 (33%)	201 (25.6%)
1001–2000 Turkish liras	313 (39.2%)	294 (37.5%)
2001–5000 Turkish liras	160 (20.1%)	139 (17.7%)
Financial aid (yes), *n* (%)	157 (19.7%)	120 (15.3%)
Legal status, *n* (%)		
Temporary protection status	713 (89.3%)	Not applicable
Asylum seeker	22 (2.8%)	433 (55.2%)
Conditional refugee	16 (2%)	71 (9%)
Residency permit	9 (1.1%)	138 (17.6%)
Turkish nationality	27 (3.4%)	9 (1.1%)
No legal status	11 (1.4%)	134 (17%)
Education level, years: mean (s.d.)	9.20 (4.24)	11.35 (4.24)
Length of stay, months: mean (s.d.)	69.05 (26.35)	36.32 (24.43)
Potentially traumatic incidents, mean (s.d.)	3.86 (3.19)	5.56 (3.70)
Socioeconomic displacement stressors, mean (s.d.)	1.60 (0.85)	2.12 (0.83)
Structural stressors, mean (s.d.)	1.03 (0.96)	2.06 (1.03)

### Measures

#### Harvard Trauma Questionnaire (HTQ)

Exposure to potentially traumatic events either in the home country or on the way to Türkiye was assessed by Part I of the Harvard Trauma Questionnaire (HTQ),^20^ which consists of 17 items (e.g. lack of food or water, imprisonment, serious injury) rated as 0 (no/absent) or 1 (yes/present). The total score ranges from 0 to 17. The questionnaire is one of the most used scales in conflict-affected populations, including Syrian and Afghan refugees.^21,22^ As the items are rated on a categorical scale, internal consistency was calculated using McDonald's omega,^23^ which was 0.82 for both Syrians and Afghans.

#### The Hopkins Symptom Checklist (HSCL-25)

Symptoms of depression and anxiety were measured using the Hopkins Symptom Checklist (HSCL-25).^24^ The scale has 25 items, 10 of which are for anxiety symptoms (e.g. feeling fearful, trembling) and the remaining 15 for depressive symptoms (e.g. feeling low in energy, slowed down, feeling lonely). Participants rate each item on a 4-point Likert scale ranging from 1 (not at all) to 4 (extremely). The HSCL-25 has been used before in Syrian and Afghan populations.^12,22^ Based on a previous study with refugees in Türkiye,^12^ mean cut-off scores of ≥2 for anxiety and ≥2.1 for depression were used to calculate probable anxiety and depression. These cut-off scores are more conservative than the conventional cut-off score of ≥1.75,^24^ and are less likely to provide overinflated probabilities. Confirmatory factor analysis (CFA) confirmed the originally structured two factors (the anxiety and depression subscales) for both Syrians (χ^2^(274) = 1313.209, *P* < 0.001, CFI = 0.914, RMSEA = 0.069 [90% CI 0.065–0.073], SRMR = 0.039) and Afghans (χ^2^(273) = 1323.381, *P* < 0.001, CFI = 0.905, RMSEA = 0.075 [90% CI 0.071–0.079], SRMR = 0.045). Cronbach's alphas for anxiety and depression were 0.92 and 0.93 for Syrians and 0.92 and 0.94 for Afghans.

#### The short form of the Post-Traumatic Stress Disorder Checklist for DSM-5 (PCL-5)

Post-traumatic stress disorder (PTSD) symptoms were assessed using the short form of the Post-Traumatic Stress Disorder Checklist (PCL-5),^25^ which is based on the DSM-5 diagnostic criteria for PTSD. The short form consists of four items, measuring symptoms of hyperarousal, intrusion, avoidance and negative cognition. Participants are asked to indicate to what extent they had experienced these symptoms in the past month on a 5-point Likert scale from 0 (not at all) to 4 (extremely). Higher total scores indicate higher PTSD symptoms. A cut-off score of ≥5 was used to indicate probable PTSD, as established in previous research.^26^ The Arabic and Farsi version of the PCL-5 have been validated and used before.^27,28^ Cronbach's alpha was 0.80 for Syrians and 0.83 for Afghans.

#### Post-Migration Living Difficulties Checklist

The Post-Migration Living Difficulties Checklist^29,30^ was used to assess to what extent the participants had been experiencing a wide range of post-displacement stressors in the previous 12 months. It has 17 items rated on a 5-point Likert scale ranging from 0 (not a problem) to 4 (very serious problem). The scale has been widely used in conflict-affected populations, including Syrian refugees.^31^ Following the categorisation by Li et al,^32^ we used CFA to test the factor resolution of the scale in terms of socioeconomic, social and interpersonal factors and asylum process-related post-displacement difficulties. However, the model did not fit the data well for the Syrian and Afghan samples. We therefore combined socioeconomic and interpersonal dimensions as socioeconomic post-displacement difficulties (12 items) (sample item: discrimination, separation from family, difficulties with employment, difficulties with financial assistance) and also reworded the asylum process-related difficulties as structural post-displacement difficulties (5 items) (sample item: unable to return home in emergency, difficulties with asylum procedures, not being recognised as a refugee). This two-factor structure of the scale fit the data well for both samples (χ^2^(111) = 593.636, *P* < 0.001, CFI = 0.909, RMSEA = 0.074 [90% CI 0.068–0.079], SRMR = 0.056 for the Syrian sample; χ^2^(114) = 502.979, *P* < 0.001, CFI = 0.901, RMSEA = 0.069 [90% CI 0.063–0.075], SRMR = 0.050 for the Afghan sample). Cronbach's alphas for socioeconomic and structural post-displacement difficulties were 0.87 and 0.78 for Syrians and 0.84 and 0.77 for Afghans.

### Statistical analyses

Confirmatory factor analyses to test the factor structures of the measures were conducted in Mplus 8.5.^33^ Descriptive analyses and hypothesis testing were conducted in SPSS Version 26.^34^ Descriptive statistics were performed to show the sociodemographic characteristics of both the Syrian and Afghan samples. Mean differences on exposure to potentially traumatic events and post-displacement stressors across gender were tested through one-way ANOVA and across refugee groups they were tested through one-way ANCOVA. The normality assumption was tested on a normal Q–Q plot with checking of skewness (which ranged from 0.99 to −0.41) and kurtosis values (ranging from 0.47 to −0.38).

Three binary mental disorder variables (depression, anxiety and PTSD) were calculated on the basis of the cut-off scores in previous studies. Logistic regression was conducted to test the differences between Syrians and Afghans in terms of the prevalence of the mental disorders. To identify the risk factors for the mental disorders, multiple logistic regression was performed for both Syrians and Afghans. Sociodemographic variables (e.g. age, gender, marital status, income level, education in years and length of stay) and potentially traumatic events and socioeconomic and structural displacement stressors were included in the logistic regression as the potential explanatory variables for the three probable mental disorders. The included predictor variables were checked for multicollinearity and influential outliers using scatterplots.

## Results

### Exposure to potentially traumatic events and post-displacement stressors

We found that 72.7% (580) of Syrian and 84.6% (664) of Afghan participants reported at least two potentially traumatic events in their home country or en route to Türkiye. In both groups, the most frequently reported potentially traumatic event was ‘living in a combat situation’ (72.7% for Syrians, 66.4% for Afghans), followed by ‘lack of food or water’ (56% for Syrians, 56.1% for Afghans). There was no statistically significant mean difference between females and males in experiencing potentially traumatic events (*F*(1,781) = 0.013, *P* = 0.909) among Syrian participants. In contrast, Afghan males reported a significantly higher number of traumatic events than Afghan females (*F*(1,773) = 4.92, *P* = 0.027). Overall, Afghan participants reported a significantly higher number of traumatic events than Syrian participants (*F*(1,1555) = 79.93, *P* < 0.001) after adjustment for gender. There was no statistically significant mean difference between females and males in terms of experiencing displacement stressors (*F*(1,781) = 0.29, *P* = 0.593) in the Syrian and Afghan samples (*F*(1,703) = 0.06, *P* = 0.815). Overall, Afghan participants reported a significantly higher level of socioeconomic and structural displacement stressors than Syrian participants (*F*(1,1485) = 133.36, *P* < 0.001 and *F*(1,1485) = 352.49, *P* < 0.001 respectively) after adjustment for gender.

### Prevalence of common mental disorders

The prevalence of probable depression, anxiety and PTSD were 41.1%, 39.6% and 41.6% respectively for Syrians and 50.3%, 42% and 46.5% respectively for Afghans. Among Syrian participants, 56.7% suffered from at least one disorder, 41.0% from at least two disorders and 26.4% from all three disorders. Among Afghan participants, 67.8% had at least one disorder, 53.7% had more than one disorder and 37.7% had all three disorders. Even after adjustment for gender, Afghan participants were more likely than Syrians to have probable depression (OR = 2.21 [95% CI 1.77–2.75]), anxiety (OR = 1.58 [95% CI 1.27–1.96]) and PTSD (OR = 1.74 [95% CI 1.40–2.17]). After controlling for potentially traumatic events and displacement-related stressors, the only significant difference between Afghans and Syrians was the probable rate of anxiety, with Afghans having a higher risk than Syrians (OR = 1.54 [95% CI 1.16–2.06]).

### Risk factors associated with common mental disorders

Holding all other variables constant, there was a strong association between gender and the three mental disorders among Syrian participants. Females were almost twice as likely to have probable depression (OR = 2.19 [95% CI 1.48–3.25]), anxiety (OR = 1.97 [95% CI 1.33–2.91] and PTSD (OR = 1.75 [95% CI 1.19–2.56]). As the education level increased, the probability of having depression (OR = 1.05 [95% CI 1.01–1.10]), anxiety (OR = 1.06 [95% CI 1.01–1.11]) and PTSD increased too (OR = 1.07 [95% CI 1.02–1.11]). In addition to demographic characteristics, exposure to potentially traumatic events was significantly associated with the increased risk of having mental disorders (OR = 1.14 [95% CI 1.06–1.21] for depression; OR = 1.19 [95% CI 1.12–1.28] for anxiety; OR = 1.18 [95% CI 1.11–1.27] for PTSD). Further, a higher level of both socioeconomic and structural post-displacement stressors was associated with a higher risk of anxiety (OR = 1.84 [95% CI 1.35–2.50] and OR = 1.49 [95% CI 1.16–1.93] respectively), whereas only socioeconomic post-displacement stressors were significant risk factors for depression (OR = 3.29 [95% CI 2.37–4.56]) and PTSD (OR = 2.23 [95% CI 1.64–3.04]).

For Afghan participants, female gender was associated with more than two times higher risk of having mental disorders (OR = 2.74 [95% CI 1.73–4.33] for depression; OR = 2.69 [95% CI 1.74–4.16] for anxiety; OR = 2.25 [95% CI 1.42–3.59] for PTSD). Older age significantly increased the risk of having anxiety (OR = 1.03 [95% CI 1.01–1.06]). In terms of residency status, compared with legal status holders, asylum seekers reported a significantly higher level of anxiety (OR = 2.07 [95% CI 1.05–4.09]) and PTSD (OR = 3.15 [95% CI 1.54–6.41]). Those without legal status had a higher level of depression (OR = 2.00 [95% CI 1.12–3.58]) and PTSD (OR = 3.23 [95% CI 1.77–5.89]) than those with legal status. Similar to Syrians, potentially traumatic events were associated with a heightened risk of having depression (OR = 1.17 [95% CI 1.09–1.26]), anxiety (OR = 1.19 [95% CI 1.11–1.27]) and PTSD (OR = 1.21 [95% CI 1.12–1.30]). Last, both socioeconomic and structural post-displacement stressors significantly increased the risk of having anxiety (OR = 2.20 [95% CI 1.53–3.18] and OR = 1.37 [95% CI 1.02–1.82] respectively) and PTSD (OR = 2.60 [95% CI 1.78–3.80] and OR = 1.51 [95% CI 1.12–2.04] respectively), but only socioeconomic post-displacement stressors were associated with depression (OR = 2.86 [95% CI 1.96–4.16]). Tables 2 and 3 shows the results of multiple logistic regression for Syrians and Afghans respectively. Further results are shown in supplementary Tables 1–3, available at https://dx.doi.org/10.1192/bjp.2022.573.

**Table 2:** Adjusted odd ratios for rates of depression, anxiety and post-traumatic stress disorder (PTSD) among Syrian participants (*n* = 663)

Predictor variables	Depression		Anxiety		PTSD	
	Adjusted OR (95% CI)	*P*	Adjusted OR (95% CI)	*P*	Adjusted OR (95% CI)	*P*
Gender (ref.: male)	2.19*** (1.48–3.25)	<0.001	1.97*** (1.33–2.91)	<0.001	1.75** (1.19–2.56)	0.004
Age	0.99 (0.98–1.01)	0.464	1.02 (0.99–1.03)	0.086	1.00 (0.98–1.02)	0.928
Marital status (ref.: unmarried)	0.69 (0.45–1.06)	0.089	0.98 (0.63–1.51)	0.911	0.97 (0.63–1.49)	0.878
Education level (in years)	1.05* (1.01–1.10)	0.025	1.06* (1.01–1.11)	0.021	1.07** (1.–1.11)	0.007
Income (ref.: <1000 Turkish liras)						
1001–2000 Turkish liras	1.12 (0.73–1.71)	0.619	0.68 (0.44–1.04)	0.78	0.71 (0.47–1.09)	0.114
2001–5000 Turkish liras	0.67 (0.40–1.12)	0.131	0.73 (0.44–1.20)	0.214	0.69 (0.42–1.14)	0.144
Length of stay (in months)	1.00 (0.99–1.01)	0.540	1.00 (0.99–1.01)	0.283	1.00 (0.99–1.01)	0.488
Exposure to traumatic incidents	1.14*** (1.06–1.21)	<0.001	1.19*** (1.12–1.28)	<0.001	1.18*** (1.12–1.27)	<0.001
Socioeconomic displacement stressors	3.29*** (2.37–4.56)	<0.001	1.84*** (1.35–2.50)	<0.001	2.23*** (1.64–3.04)	<0.001
Structural displacement stressors	1.01 (0.78–1.30)	0.968	1.49** (1.16–1.93)	0.002	1.25 (0.97–1.62)	0.080

Ref., reference.

*P < 0.05, **P < 0.01, ***P < 0.001.

**Table 3:** Adjusted odd ratios of rates of depression, anxiety and post-traumatic stress disorder (PTSD) among Afghan participants (*n* = 584)

Predictor variables	Depression		Anxiety		PTSD	
	Adjusted OR (95% CI)	*P*	Adjusted OR (95% CI)	*P*	Adjusted OR (95% CI)	*P*
Gender (ref.: male)	2.74*** (1.73–4.33)	<0.001	2.69*** (1.74–4.16)	<0.001	2.25*** (1.42–3.56)	<0.001
Age	1.02 (0.99–1.05)	0.096	1.03* (1.01–1.06)	0.011	1.02 (0.99–1.05)	0.075
Marital status (ref.: unmarried)	0.68 (0.42–1.05)	0.083	1.10 (0.71–1.71)	0.660	0.89 (0.56–1.41)	0.623
Education level (in years)	0.99 (0.95–1.05)	0.958	0.98 (0.93–1.03)	0.404	0.98 (0.93–1.03)	0.399
Legal status (ref.: legal status holders)						
Asylum seekers	0.05 (0.88–3.46)	0.110	2.07*(1.05–4.09)	0.037	3.15** (1.54–5.41)	0.002
No legal status	2.00* (1.12–3.58)	0.019	1.73 (0.97–3.07)	0.064	3.23*** (1.77–5.89)	<0.001
Income (ref.: <1000 Turkish liras)						
1001–2000 Turkish liras	0.78 (0.48–1.27)	0.316	0.71 (0.44–1.13)	0.151	0.94 (0.57–1.53)	0.792
2001–5000 Turkish liras	0.87 (0.49–1.56)	0.645	0.79 (0.45–1.39)	0.409	0.93 (0.52–1.67)	0.806
Length of stay (in months)	1.00 (0.99–1.01)	0.675	1.00 (0.99–1.01)	0.823	1.00 (0.99-1.01)	0.928
Exposure to traumatic incidents	1.17*** (1.09-1.26)	<0.001	1.19*** (1.11-1.27)	<0.001	1.21*** (1.12–1.29)	<0.001
Socioeconomic displacement stressors	2.86*** (1.96–4.16)	<0.001	2.20*** (1.53–3.18)	<0.001	2.60*** (1.78–3.80)	<0.001
Structural displacement stressors	1.13 (0.84–1.51)	0.430	1.37* (1.02–1.82)	0.034	1.51** (1.21–2.04)	0.007

Ref., reference.

*P < 0.05, **P < 0.01, ***P < 0.001.

## Discussion

### Main findings and interpretation

The present study aimed to examine and compare the self-reported rates of depression, anxiety and PTSD among Syrian and Afghan refugees in Türkiye. We found high rates of symptoms among both groups. More than half of the participants in both groups (56.7% for Syrians and 67.8% for Afghans) reported at least one of these mental health problems. The current estimates for Syrians were higher than those reported among Syrian refugees in Türkiye and Western countries.^12,35^ However, they are comparable with the reported estimates in a recent study conducted among Syrian refugees during the COVID-19 pandemic.^15^ In that study, the prevalence rates of depression and anxiety were 51.9% and 42.9% among the participants. Thus, the observed increase in the prevalence rates might be due to the additional difficulties during the pandemic, such as lack of access to hygiene materials, losing jobs and being unable to pay rent.

Estimated prevalence rates for Afghans were higher than those reported in Western countries,^36,37^ but were similar to those reported following the conflict in Afghanistan and in neighbouring countries. Scholte et al^38^ and Cardozo et al^39^ found prevalence rates of 38.5% and 67.7% for depression, 51.8% and 72.2% for anxiety and 20.4% and 42.1% for PTSD among Afghans in different areas of Afghanistan. A systematic review reported that mental health problems are highly prevalent among Afghan refugees in Iran, with at least one-third suffering from one mental health problem.^40^

In line with our initial assumption, Afghans reported a higher number of potentially traumatic events and displacement-related stressors than Syrians. Entrenched war and conflict in Afghanistan over 40 years and the dangerous migration route from Afghanistan to Türkiye might explain the higher level of potentially traumatic events reported by Afghans. A recent in-depth study with 50 Afghans in Istanbul sheds light on the precarious conditions of Afghans before, during and after migrating to Türkiye. The travel from Afghanistan to Türkiye was often perilous and could take several months.^41^ Although both Syrians and Afghans live in the same displacement context, the degree to which they experience post-displacement difficulties evidently varies. Mental health problems, mainly anxiety, were higher among Afghans than Syrians even after controlling for the effect of traumatic events and displacement-related stressors. The disparities in post-displacement experiences and probable anxiety might be explained by legal status. Their temporary protection status opens the doors for Syrians to utilise basic services even if in practice access may still be challenging. Unlike Syrians, many Afghans live as asylum seekers waiting for the decision or as irregular migrants, which hampers their access to basic services.^4^ Within Türkiye many Afghans live under constant fear of deportation, which prompts them to live as invisibly as possible in order to stay under the radar of the authorities and obliges them accept the hardest and the most unstable jobs.^41^ The uncertainty of their legal status is likely to create hardships for them and hinder their ability to look ahead,^32^ which might explain the substantial differences in displacement experiences and mental health conditions among Afghans and Syrians in the present study.

As for the second aim of the study, we investigated the risk factors for mental health problems in both groups. Consistent with the existing evidence, female gender appeared as a significant risk factor for all three mental health problems among Syrians and Afghans.^10,12^ The higher risk for females to develop mental health problems might be explained by gender roles and experiences of gender-based violence such as intimate partner violence, early or forced marriages and social isolation.^42,43^ Further, we found that higher education level was associated with increased risk of having mental health problems among Syrians only. Since qualifications in their home country are not easily transferable to the new setting, they are likely to be either unemployed or underemployed (working below the level of education or training that they completed in Syria).^44^ Therefore, higher education level poses a profound risk for mental illness because those with higher education might have more to lose from conflict and displacement.^45^ Older age was associated with higher risk of anxiety only among Afghans. Previous studies with Afghans also showed that older Afghans have more mental health problems.^37,38^ Legal status was another important risk factor among the Afghan refugees. Compared with legal status holders, asylum-seeking applicants and those with no legal status (including those whose applications were rejected and those who did not apply for protection) reported higher levels of mental health problems. This is in line with research highlighting the adverse impacts of prolonged and uncertain asylum-seeking processes.^32,46^ Being an asylum seeker is a potent risk factor for mental illness among Afghans in Western countries^10^ and in neighbouring countries such as Iran.^40^ Even after a long process of asylum-seeking, permanent residency status is not guaranteed to the applicants. In Türkiye, the asylum-seeking process is complicated, with the possibility of withdrawal of the residency if the home country becomes safe to return to, which hinders individual's ability to look ahead and adapt to the new life.^32^ This situation, unfortunately, puts Afghans in an even more vulnerable position.

Our finding showing that exposure to potentially traumatic events increases the risk of having common mental health problems dovetails with the large body of research among forcibly displaced communities.^17,47^ As for the post-displacement stressors, we found that socioeconomic displacement stressors (e.g. discrimination, isolation, financial difficulties) were a more consistent predictor of mental health problems among Syrians and Afghans. Those who experienced higher levels of socioeconomic stressors were two to three times more likely to develop mental health problems. Compared with traumatic events, this increased risk might be related to the characteristics of socioeconomic stressors that are proximal in time and ubiquitous in daily life.^48^ Structural displacement stressors were associated with higher risk of anxiety among both Syrians and Afghans and higher risk of PTSD among Afghans. Considering the nature of structural stressors, such as difficulties related to the asylum process and being fearful of being sent back to the country of origin, it is imperative to assume that these stressors fuel a feeling of uncertainty, which provides grounds for anxiety and related disorders.^49^ These findings are of particular importance in showing that stressors encountered in different areas of life affect mental health to a different degree.^13^

### Strengths, limitations and implications

To the best of our knowledge, this study is the first to provide a comparative picture of the hardships of two major refugee groups in Türkiye. Since the onset of the Syrian war, there have been several studies investigating the mental health conditions of Syrians in the neighbouring and Western countries.^35,50^ Several international collaborations are undertaking studies testing the effectiveness of psychosocial interventions on mental health among Syrians.^21,51^ However, despite the conflict in their country and continuing displacement, Afghans’ displacement conditions and mental health have received relatively scant attention. With the withdrawal of foreign military forces and Taliban takeover of the country, the humanitarian situation in Afghanistan further deteriorated, putting approximately 25 million people in need. Owing to the dire conditions in Afghanistan – the conflict, natural disasters, the COVID-19 pandemic, economic collapse – almost 1 million people were displaced inside the country and thousands of them sought refuge in Iran and Pakistan,^52^ with the possibility of many more people fleeing the country in the near future. It is likely therefore that more Afghans will find their way to Türkiye to either stay or pass through to European countries. We hope that the findings of the high mental health needs of Afghans in Türkiye will lead to more academic work on this group and to improved humanitarian programming.

Our findings reveal the impacts of potentially traumatic conflict-related events and post-displacement stressors, highlighting vulnerable groups among Syrians and Afghans, such as females, the highly educated and older individuals. Although resources might be limited to directly working with the traumatic events, especially in low- and middle- income countries that are short of mental health resources,^53^ efforts might be spent on improving life circumstances for refugees and asylum seekers living in displacement through, for example, less restrictive asylum policies, better access to education and the labour market and cash-based interventions.^48^ Further, our findings suggest that socioeconomic stressors might be more proximal and pervasive than structural ones, further increasing susceptibility to mental health problems. We assume therefore that structural barriers or stressors influence mental health via precipitating socioeconomic stressors, but this assumption requires further inquiry.

The present study has number of limitations. First, owing to the COVID-19 pandemic, we conducted online surveys, which might restrict our ability to extrapolate the current findings to Syrians and Afghans in Türkiye in general. Second, this study was based on self-report of mental health problems. A recent systematic review and meta-analysis showed that self-report measurements might yield overestimated prevalence rates.^16^ Therefore, it is important to be cautious in making inferences about the prevalence rates of common mental health problems among Syrians and Afghans in Türkiye. Further, we utilised the most commonly used measures to assess mental health problems among these groups. Consequently, we might have overlooked idiosyncratic ways of expressing mental health problems.^54^

## Data Availability

The data that support the findings of this study are available from the corresponding author (C.A.) on reasonable request.

## Supplementary material

For supplementary material accompanying this paper visit http://doi.org/10.1192/bjo.2022.573. click here to view supplementary material
