# Supplementary material for: Estimated prevalence rates and risk factors for common mental health problems among Syrian and Afghan refugees in Türkiye
Source: BJPsych Open. 2022 Sep 15;8(5):e167. doi: 10.1192/bjo.2022.573 (PMC9534906; doi:10.1192/bjo.2022.573)
Supplement: Supplementary file 1 [file S2056472422005737sup001.docx]

**Supplementary Materials**

**Table S1.** Logistic regression results for the comparison between Syrians and Afghans on probable depression, anxiety, and PTSD.

| Predictor variables | Depression |  | Anxiety |  | PTSD |  |
| --- | --- | --- | --- | --- | --- | --- |
|  | Adjusted OR (95% CI) | p values | Adjusted OR (95% CI) | p values | Adjusted OR (95% CI) | p values |
| Group (ref=Syrians) | 1.91***(1.55-2.34) | <.001 | 1.39 (1.13-1.71) | .002** | 1.57***(1.27-1.93) | <.001 |

**p* < 0.05, **p* < 0.01, ****p* <0.001.

**Table S2.** Logistic regression results for the comparison between Syrians and Afghans on probable depression, anxiety, and PTSD, adjusted for gender

| Predictor variables | Depression |  | Anxiety |  | PTSD |  |
| --- | --- | --- | --- | --- | --- | --- |
|  | Adjusted OR (95% CI) | p values | Adjusted OR (95% CI) | p values | Adjusted OR (95% CI) | p values |
| Group (ref=Syrians) | 2.21***(1.77-2.75) | <.001 | 1.58***(1.27-1.96) | <.001 | 1.74***(1.40-2.17) | <.001 |
| Gender (ref=male) | 1.80***(1.44-2.24) | <.001 | 1.76***(1.41-2.18) | <.001 | 1.61***(1.30-2.00) | <.001 |

**p* < 0.05, **p* < 0.01, ****p* <0.001.

**Table S3.** Logistic regression results for the comparison between Syrians and Afghans on probable depression, anxiety, and PTSD, after adjusted for conflict-related traumatic experiences and post-displacement stressors.

| Predictor variables | Depression |  | Anxiety |  | PTSD |  |
| --- | --- | --- | --- | --- | --- | --- |
|  | Adjusted OR (95% CI) | p values | Adjusted OR (95% CI) | p values | Adjusted OR (95% CI) | p values |
| Group (ref=Syrians) | 1.15(.87-1.53) | .326 | 1.54**(1.16-2.06) | .003 | 1.33(1.00-1.78) | .052 |
| Gender (ref=male) | 2.25***(1.73-2.93) | <.001 | 2.25***(1.74-2.92) | <.001 | 2.01***(1.55-2.61) | <.001 |
| Exposure to traumatic incidents | 1.16***(1.11-1.21) | <.001 | 1.18***(1.13-1.23) | <.001 | 1.17***(1.13-1.23) | <.001 |
| Socio-economic displacement stressors | 3.32***(2.67-4.13) | <.001 | 2.30(1.87-2.83) *** | <.001 | 2.73***(2.21-3.38) | <.001 |
| Structural displacement stressors | 1.00(.85-1.19) | .968 | 1.29**(1.10-1.52) | .002 | 1.19*(1.01-1.41) | .041 |

**p* < 0.05, **p* < 0.01, ****p* <0.001.
